# A study protocol for improving the delivery of acute kidney replacement therapy (KRT) to critically ill patients in Alberta – DIALYZING WISELY

**DOI:** 10.1186/s12882-022-02990-6

**Published:** 2022-11-16

**Authors:** Dawn Opgenorth, Sean M. Bagshaw, Vincent Lau, Michelle M. Graham, Nancy Fraser, Scott Klarenbach, Louise Morrin, Colleen Norris, Neesh Pannu, Selvi Sinnadurai, Shelley Valaire, Xiaoming Wang, Oleksa G. Rewa

**Affiliations:** 1 Department of Critical Care Medicine, Faculty of Medicine and Dentistry, University of Alberta and Alberta Health Services, CSB 2-124 8440 112th St NW, Edmonton, Alberta T6G 2B7 Canada; 2 Critical Care Strategic Clinical Network, Alberta Health Services, Edmonton, Alberta Canada; 3 Department of Medicine, Faculty of Medicine and Dentistry, University of Alberta, Edmonton, Canada; 4 Cardiovascular Health and Stroke Strategic Clinical Network, Alberta Health Services, Edmonton, Alberta Canada; 5 Medicine Strategic Clinical Network, Alberta Health Services, Edmonton, Alberta Canada; 6 Maternal, Neonatal, Child and Youth Strategic Clinical Network, Alberta Health Services, Edmonton, Alberta Canada; 7 Health Services Statistical and Analytic Methods, Alberta Health Services, Edmonton, Alberta Canada

**Keywords:** Critical care medicine, Intensive care, Acute renal replacement therapy, Care pathway integration

## Abstract

**Background:**

Acute kidney replacement therapy (KRT) is delivered to acutely ill patients to support organ function and life in the Intensive Care Unit (ICU). Implementing standardized acute KRT pathways can ensure its safe and effective management. At present, there is no standardized approach to the management of acute KRT in Alberta ICUs.

**Methods:**

Dialyzing Wisely is a registry embedded, stepped-wedge, interrupted time-series evaluation of the implementation of a standardized, stakeholder-informed, and evidence-based acute KRT pathway into Alberta ICUs. The acute KRT pathway will consist of two distinct phases. First, we will implement routine monitoring of evidence-informed key performance indicators (KPIs) of acute KRT. Second, we will provide prescriber and program reports for acute KRT initiation patterns. After the implementation of both phases of the pathway, we will evaluate acute KRT performance quarterly and implement a customized suite of interventions aimed at improving performance. We will compare this with baseline and evaluate iterative post implementation effects of the care pathway.

**Discussion:**

Dialyzing Wisely will implement, monitor, and report a suite of KPIs of acute KRT, coupled with a care pathway that will transform the quality of acute KRT across ICUs in Alberta. This program will provide a framework for scaling evidence-informed approaches to monitoring and management of acute KRT in other jurisdictions. We anticipate improvements in acute KRT performance, decreased healthcare system costs and improved patient quality of life by decreasing patient dependence on maintenance dialysis.

**Trial registration:**

Clinicaltrials.gov, NCT05186636. Registered 11, January, 2022.

**Supplementary Information:**

The online version contains supplementary material available at 10.1186/s12882-022-02990-6.

## Background

Acute kidney replacement therapy (KRT) is a core life support technology used in approximately 10–12% of critically ill patients [1]. It is generally used to support patients with overt kidney failure or as part of a broader strategy for multi-organ support in ICU settings, along with mechanical ventilation and vasoactive medications. Its utilization has expanded, with recent estimates showing growth of more than 10% per year [2, 3]. Acute KRT in the ICU can be delivered intermittently (i.e., intermittent kidney replacement therapy [IKRT]) or continuously (i.e., continuous kidney replacement therapy [CKRT]) depending on the available technology.

### Acute KRT in Alberta

In 2019, acute KRT was initiated in 1278 patients across 18 adult ICUs in Alberta, which represents 5–8% of our critically ill population. This coincides with 3278 KRT patient-days of continuous KRT and 398 intermittent KRT patient-days with estimated direct healthcare costs of $764–865 per day and $528 per day, respectively [3, 4]. Non-adherence to evidence-based practice may have led to an additional 56 patients per year with new end stage kidney disease (ESKD) [5]. These survivors of critical illness complicated by severe AKI, who are now receiving maintenance dialysis after hospital discharge, have attributable healthcare costs exceeding $100,000 annually per patient [6, 7]. These costs do not include associated travel and lost work time costs as well as significant impairments in the quality of lives of these patients. Strategies are needed to standardize and reduce variations in care, improve patient centered outcomes, improve health system efficiencies and reduce patient and health care system costs.

### Current acute KRT practices are not standardized or monitored in Alberta

Presently in Alberta, the provision of acute KRT occurs without routine capture and reporting of performance indicators, and only the number of patients and patient-days receiving acute KRT are routinely reported [8]. Without mechanisms in place to better monitor this therapy, healthcare professionals cannot appreciate whether the therapy they provide aligns with current evidence and best practices and whether adjustments can be made to their practice to improve delivery of KRT [9]. This in turn, contributes to suboptimal, less effective, and potentially costlier provision of acute KRT through systemic inefficiencies, increased resource use, and higher healthcare professional workload for potentially lower value therapy.

### New evidence-based care practices are not always integrated into routine care

Care practices evolve as new evidence on best practice emerges. However, it is generally recognized that in the absence of targeted strategies, there is a delayed uptake of evidence into practice by at least 5–10 years [10]. Utilizing the principles of a learning health care system in which 1) knowledge gaps and variations in practice are identified; 2) clinical research is integrated into routine bedside care to address knowledge gaps; and 3) results are then seamlessly implemented into practice; would significantly decrease the knowledge to action gaps in the integration of new evidence-based practices into current acute KRT [11].

#### Monitoring of KRT practices

Evidence shows that monitoring and reporting of KPIs is an important aspect of any high performing acute KRT program [12]. Previous work by our study team has identified, validated and prioritized KPIs for acute KRT care [1, 2]. In addition, others have shown that initiatives such as: the implementation of KPIs through a quality dashboard to measure adherence to KRT standards; establishment of evidence-informed benchmarks; enhancement of documentation templates and acute KRT provider education; and integration of an evidence-based quality improvement system to support the management of KRT, have been successful in improving the quality of KRT delivery and establishing infrastructure to ensure ongoing sustainability of quality initiatives [13–15].

#### Time of KRT initiation

The STARRT-AKI trial found that standard initiation of acute KRT was not associated with increased mortality. However, accelerated initiation was found to lead to a higher occurrence of adverse events and a 74% relative increase in risk of failure to recover kidney function and remain on maintenance KRT at 90-days when compared to the standard delayed initiation strategy [5]. While these findings were published in 2020, there has been no formal process through which to integrate them into clinical practice in Alberta ICUs.

The implementation of the Dialyzing Wisely evidence-based care pathway would provide a standardize means of integrating new and evolving evidence-informed best care practices. In addition, practitioners would have a standard mechanism through which to improve practice utilizing strategies such as audit and feedback, benchmarking and interactive peer group learning.

## Objectives

**Fig. 1:**
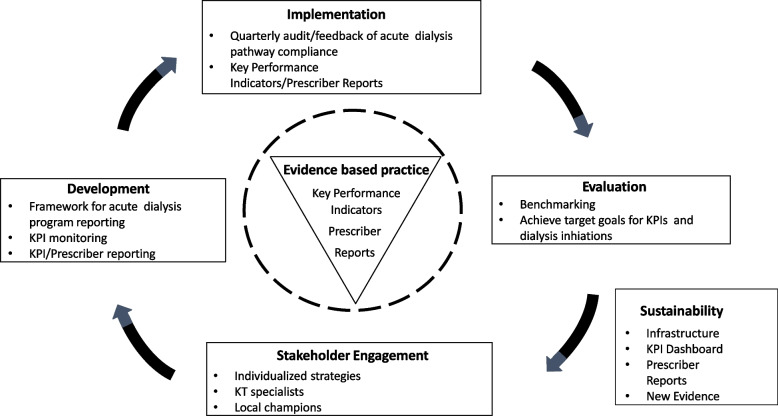
Care Pathway for Dialyzing Wisely

The primary objective of this project is to implement and evaluate a multifaceted evidence-informed care pathway into acute KRT programs in Alberta ICUs (Fig. 1).

### Research questions

1. Can we develop and implement a standardized framework to be implemented in all acute KRT programs?
2. Will monitoring the performance of our acute KRT delivery by means of evidence-based KPIs result in improved performance and a decrease in acute KRT program costs?
3. Can we rapidly implement the findings of novel programs of research into clinical practice to improve patient-centered outcomes and decrease both short and long-term healthcare costs?

## Methods/design

**Fig. 2:**
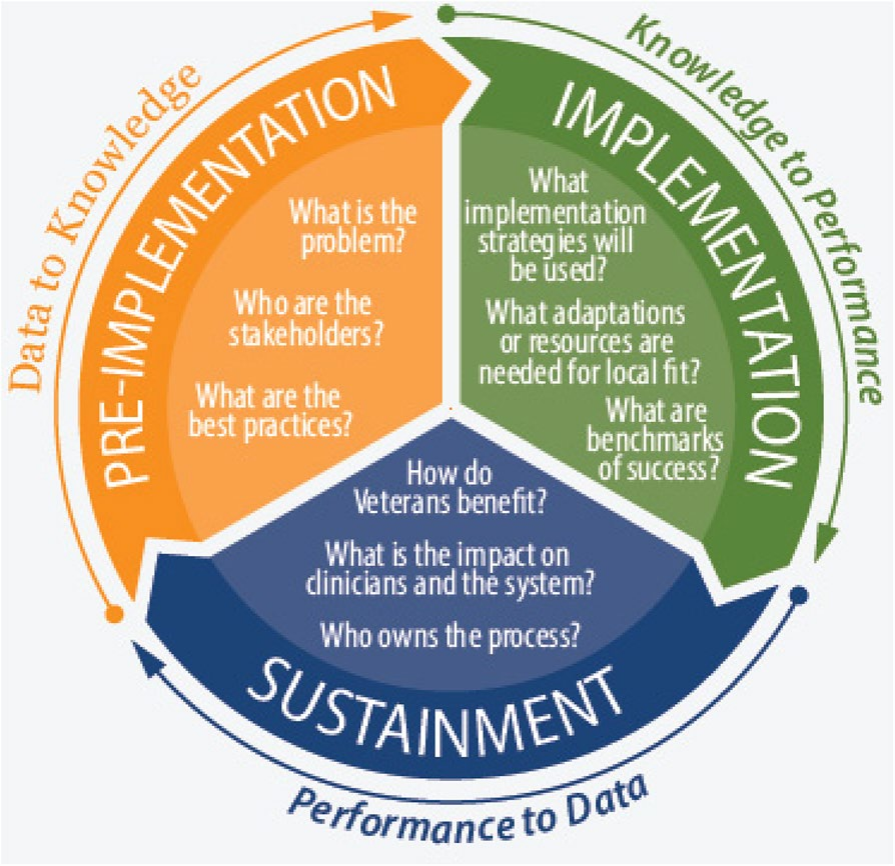
Outline of learning health system knowledge to action framework. The healthcare system data are used to set local priorities for improvement. Data on priority problems and potential contributors help find the evidence based solutions to improve local health systems (data to knowledge). Using evidence based knowledge, this will inform local quality improvement and implementation science guided efforts (knowledge to performance). Finally, the continuous monitoring of local KPIs and practices will develop practice based knowledge while revealing future opportunities for quality improvement (performance to data). Adapted from Kilbourne et al. [16]

Dialyzing Wisely is a multi-centre, registry embedded, stepped-wedged, interrupted time-series evaluation of the implementation of an evidence-based and best practice acute KRT pathway in the 15 adult general and cardiac ICUs and 3 pediatric general and cardiac ICUs in Alberta that provide acute KRT (Table 1). This implementation plan will follow the principles of the Learning Health System Knowledge to Action Framework (Fig. 2) [16].

**Table 1:** Alberta ICUs Delivering KRT

Site	City/Zone^**a**^	ICU Type	Hospital Type	Beds
University of Alberta Hospital General Systems ICU	Edmonton	Mixed	Academic	32
Mazankowski Alberta Heart Institute Cardiovascular ICU	Edmonton	Cardiac surgery	Academic	24
Mazankowski Alberta Heart Institute Cardiac ICU	Edmonton	Cardiac	Academic	8
Royal Alexandra Hospital ICU	Edmonton	Mixed	Academic	25
Grey Nuns Hospital ICU	Edmonton	Mixed	Community	8
Misericordia Hospital	Edmonton	Mixed	Community	10
Sturgeon Hospital ICU	Edmonton	Mixed	Community	5
Stollery Children’s Hospital Pediatric ICU	Edmonton	Mixed	Academic	16
Stollery Children’s Hospital Pediatric Cardiac ICU	Edmonton	Cardiac	Academic	16
Foothills Medical Centre ICU	Calgary	Mixed	Academic	28
Foothills Medical Centre Cardiovascular ICU	Calgary	Cardiac surgery	Academic	16
Foothills Medical Centre Cardiac ICU	Calgary	Cardiac	Academic	18
Peter Lougheed Centre ICU	Calgary	Mixed	Academic	18
Rockyview General Hospital ICU	Calgary	Mixed	Community	10
South Health Campus ICU	Calgary	Mixed	Community	10
Alberta Children’s Hospital Pediatric ICU/PCICU	Calgary	Mixed	Academic	15
Red Deer Regional Hospital ICU	Red Deer/Central	Mixed	Regional	12
Chinook Regional Hospital ICU	Lethbridge/South	Mixed	Regional	7

Edmonton zone provides acute KRT to North Zone patients as there are no KRT programs currently operating in North Zone ICU

^a^Alberta Health Service (AHS) is organized into five geographic zones: North (pop. 480,924), Edmonton (pop. 1,422,009), Central (pop. 476,6774), Calgary (pop. 1,710,560) and South (pop. 311,514) https://www.albertahealthservices.ca/assets/about/publications/ahs-ar-2020/zones.html

**Fig. 3:**
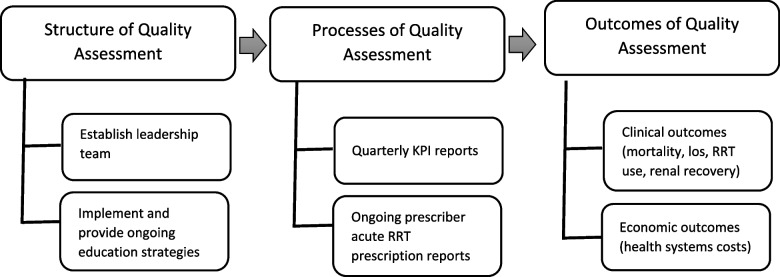
Outline of Structure-Process-Outcome framework. The framework of elements which constitute each standardized acute dialysis program is depicted above. While themes remain consistent between programs, each program may use specific elements which work best within their own processes

Dialyzing Wisely will utilize a structure-process-outcome framework for quality assessment by implementing a standardized framework to each acute KRT program (Fig. 3) [17, 18]. Each participating ICU will be structured to be led by a team with expertise in KRT (e.g., physician, educator, registered nurse, administrator) who will undergo and further disseminate targeted education strategies prior and during the intervention. Each ICU will receive quarterly performance reports on a minimum suite of essential KPIs tailored to their unit’s specific practice. Through this feedback the team members will work on adjusting and improving KRT practices that do not meet KPI benchmarks. Further, acute KRT prescribers will receive individualized reports outlining their prescription practices and their alignment with best evidence. Follow up with individual ICUs and practitioners will occur to better understand and evaluate prescribing practices. After completion of the intervention period, the impact of the intervention will be measured through select clinical and economic outcome measures (e.g., mortality, lengths of stay, KRT utilization and renal recovery). At this time, using our partners (outlined below) we will transition ownership to local stakeholders.

The SPIRIT checklist is available as an Additional File.

### Trial oversight

Dialyzing Wisely will be governed by an Executive Committee. The Executive Committee will be comprised of Leads from 4 participating Strategic Clinical Networks (SCNs) (Critical Care, Medicine (Kidney Health Section), Cardiovascular Health and Stroke, and Maternal, Newborn, Child and Youth). SCNs are province-wide integrated teams that collaboratively identify and solve challenges within their specific area of health. SCN membership includes clinicians, patients, operational leaders, researchers, community and industry partners and other stakeholders [19].

The Executive Committee will be supported by an International Advisory Panel. This panel will be made up of critical care nephrology experts with a specific interest in quality and safety for acute KRT. The International Advisory Panel will provide a high-level review of program performance and will provide unbiased recommendations to ensure ongoing advancement and program success.

The program will also have a Steering Committee with representation from all stakeholder groups (i.e., physicians, nurses, educators, administrators, operational leads, epidemiologists, health economists, informatics specialists and patient-partners). This Steering Committee will review KPI reports and study developments to ensure ongoing appropriate program advancement and oversight.

Finally, key stakeholders have been identified at each individual study site to operationalize and champion the implementation of the acute KRT pathway and enact change. We will also include provincial organizations to facilitate our audit and feedback process and transition ownership of the program to local sites. This will include members listed above as well as the Alberta Medical Association Physician Learning Program (PLP).

**Fig. 4:**
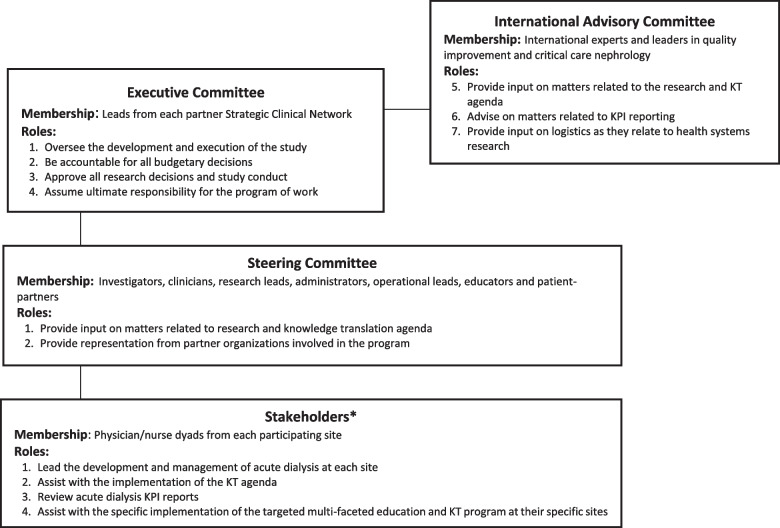
Project governance and roles structure. ^*^Stakeholders will fill the role of Leadership and Educational leads for each Acute Dialysis program, fulfilling the Structure KPI Requirements in Dialyzing Wisely. All project committees will be supported by the Program Manager and Research Assistant. Key reporting and educational messaging will be facilitated by Knowledge Translation Specialists

All aspects of the governance will be overseen by the program manager, and facilitated by the research assistant. Key reporting and educational messaging will be delivered by Clinical Practice Leads with expertise in knowledge translation strategies (Fig. 4).

### Population and eligibility

The inclusion criteria will be critically ill patients (i.e., adults and children) receiving acute KRT as part of their routine ICU care. No exclusion criteria will be applied.

### Interventions, duration and frequency of follow-up

The acute KRT pathway will consist of two specific interventions:

1. Monitoring, reporting and audit of acute KRT KPIs.
2. Provision of individualized prescriber and program reports for acute KRT initiation patterns.

The acute KRT care pathway will be implemented in a stepwise fashion with a pilot followed by randomized stepped wedge roll out at centres across Alberta over the subsequent 21 months. Roll out of ICUs will be performed in clusters and will coincide with individual site activation of a novel, province-wide electronic provincial clinical information system (CIS), Connect Care (EPIC, Verona WI).

#### Monitoring and reporting of acute KRT KPIs

The monitoring, reporting and audit of acute KRT KPIs will be done by means of automatically generated reports delivered directly to KRT stakeholders at each ICU at quarterly intervals (Table 2). These unit-level aggregate summaries of KPIs will be benchmarked to other ICUs in Alberta and will be delivered in an electronic fashion. KPIs captured in the reports for CKRT will include: 1) filter life, 2) downtime, 3) delivered dose, 4) ultrafiltration realized and 5) number of access alarms; and for IKRT: 1) treatment completion, 2) delivered dose, 3) solute clearance, 4) ultrafiltration realized and 5) catheter malfunction.

**Table 2:** Intervention definitions and parameters - Key Performance Indicators (KPIs)

**CRRT KPI**	**Operational Definition**	**Proposed Benchmark**
Filter Life	Average number of hours of filter life of all filters per quarter	> 50% of filters last 72 hours
Downtime	Time CRRT not running per day/ Each day of CRRT prescription	< 15%
Delivered Dose	Actual delivered dose in ml/Kg/h / Prescribed dose in ml/Kg/h	> 85% of dose and between 25 and 30 ml/Kg/h
Ultrafiltration Realized	Ultrafiltration realized per 24 hours/ Ultrafiltration prescribed per 24 hours	> 85%
Access Alarms	Number of alarms recorded per machine per day of therapy	< 5 alarms/d
**IRRT KPI**	**Operational Definition**	**Proposed Benchmark**
Treatment Completion	Number of IRRT treatment completed/ Total number of IRRT treatments	100%
Treatment Time	[Delivered – Prescribed hours of dialysis therapy]/ Prescribed hours of dialysis therapy	> 85% of time
Solute Clearance	Percentage difference between serum urea pre-treatment and serum urea post treatment	> 10% decrease/treatment
Ultrafiltration Realized	Ultrafiltration realized for treatment/ Ultrafiltration prescribed per treatment	> 85%
Catheter Malfunction	Number of IRRT runs with catheters reversed/ Number of IRRT runs	< 20%
**Prescriber Report Metrics**		
**RRT Initiation Criteria**		**Benchmarks**^**a**^
Serum potassium		> 6.0 mmol/L
pH		< 7.20
Serum bicarbonate		< 12 mmol/L
Oxygenation status		Impaired oxygenation as per P/F ratio of < 200
Cumulative volume status		Cumulative Fluid Balance defined as > 10% positive fluid balance^b^ anchored from time of ICU admission

^a^Benchmarks obtained from STARRT-AKI inclusion criteria

^b^Cumulative Fluid Balance is calculated as (fluid intake – fluid output in liters since ICU admission) / (weight in kilograms) × 100%

#### Prescriber reporting

An ICU-specific prescriber and program report will be provided to each prescriber and ICU on a quarterly basis. The report will include: 1) the number of acute KRT initiations, 2) acute KRT initiations based on conventional indications (i.e., hyperkalemia, acidosis, metabolic status, oxygenation status and cumulative fluid status) termed ‘appropriate initiations’, 3) general guidelines of KRT initiation criteria as per STARRT-AKI standard initiation arm protocol. (Table 2) [5] These will be benchmarked against prescriber patterns from providers in the same ICU, as well as similar ICUs across Alberta.

### Stakeholder education

Prior to implementation of the reports, each ICU will receive education strategies specifically tailored to their site. The education strategies will be informed by local acute KRT leaders, champions, and stakeholders and will serve to identify barriers and facilitators to the program (Table 3). Initial education strategies will likely contain similar themes across all sites and will be managed by our study team, however after receiving feedback through the KPI reports, each site will be encouraged to facilitate and conduct their own audit and educational activities to address any unit specific shortcomings identified in their acute KRT KPI performance.

**Table 3:** Components of the multi-faceted intervention and knowledge implementation strategy

Strategy	Description
Education	• Site grand rounds and inter-professional seminars • Monthly video/teleconferencing sessions • Site specific educational sessions by inter-professional content experts and local champions • Provide a summary of current guidelines and best practice • Development of website for repository of evidence supporting implementation including banked webinar of project • In-person or virtual visits with ICU leadership, champions and investigator teams
Coaching	• Provide ongoing resources for interpretation of KPI reports • Common troubleshooting advice cards • Provide clinical decision support resources
Audit and Feedback	• Baseline and monthly reports of process of care indicators of implementation of the intervention • Comparative performance relative to peer ICUs across province • Quarterly video/teleconferencing sessions to discuss provincial KPI reports
Reminders	• Promotional items (posters; bulletins) • Weekly electronic communication to local site champions to ensure ongoing review of KPI reports and access to additional resources

Select outlying prescribers will be contacted by the study team to further evaluate either well performing or poorly performing prescriber patterns as they relate to most recent evidence.

### Primary outcomes

- Measurement of change across acute KRT KPIs that will include the following:
  - ∘ CKRT: 1) filter life, 2) downtime, 3) delivered dose, 4) ultrafiltration realized and 5) number of access alarms
  - ∘ IKRT: 1) treatment completion, 2) delivered dose, 3) solute clearance, 4) ultrafiltration realized and 5) catheter malfunction
- Number of appropriate acute KRT initiations
- Number of patients entering maintenance KRT programs
- Acute KRT and healthcare systems costs

### Secondary outcomes

- Length of KRT
- ICU and hospital lengths of stay
- ICU and 90-day mortality
- Rates of KRT dependence at 90 days
- Health-related quality of life measurement (i.e., EQ-5D-5L and PedsQL) and patient-related outcome measures (PROMs) (i.e., ESAS-r and IPOS-renal)

### Data management

Effects of prescriber and acute KRT program patterns in the initiation of acute KRT will be determined by monitoring resource use associated with initiation of KRT at both a program level and healthcare system level. This will be done by determining first the units of each resource and then by assigning costs to each unit. At the program level we will capture the number of acute KRT initiations as well as total patient-days of acute KRT per specific modality (i.e., intermittent or continuous). These acute KRT initiations will also be adjusted for severity of illness to enable translation across units with varying case-mix, acuity and workload. Acute KRT disposable costs will include KRT filters, catheters, replacement and anticoagulation solutions.

New intake of critically ill survivors with severe AKI into ESKD dialysis programs will be reviewed on a quarterly and yearly basis to determine any changes relevant to use of this resource to the Alberta healthcare system. We will ensure that any changes in intakes reflect acute KRT initiations based on adherence to best-evidence practices.

Data will be collected on patient characteristics: (i.e., demographics, type of admission [medical, surgical, trauma]), clinical status (i.e., comorbid diseases including chronic kidney disease, primary diagnosis), acuity (i.e., APACHE II, SOFA, CFS), ICU treatment (i.e., duration of renal replacement therapy, mechanical ventilation, vasoactive therapy), ICU and hospital lengths of stay, and outcomes (i.e., renal recovery and mortality); and KRT-associated resource data: (i.e., filter use, prescription/dose, machine alarms/down time, anticoagulation, re-hospitalizations, progression of renal disease). Data variables to be captured are summarized in Table 4.

**Table 4:** Data variables

Data Variable	Description
ICU location	admission ICU
Age	years
Sex:	M/F
Weight	kg
Date of Hospital Admission	dd/mm/yyyy
Date of ICU Admission (dd/mm/yyyy):	dd/mm/yyyy
Admission class	med/surg/neuro/trauma
ICU discharge location	unit/hospital
ICU Admission Diagnosis – cardiovascular, respiratory, gastrointestinal, genitourinary/renal, endocrinological/metabolic, neurological, trauma, burn, sepsis, surgery	yes/no
Co-morbidities – AIDS, chronic RRT, chronic heart failure, respiratory insufficiency, cirrhosis, diabetes mellitus, hepatic failure, immune suppression, leukemia, lymphoma, metastatic cancer, coronary artery disease	yes/no
Clinical Frailty Scale	number
APACHE II Score	number
SOFA score	number
Invasive/non-invasive ventilation	hrs/min
Vasopressors (include type)	hrs/min
CRRT/IHD/SLED	hrs/min
Cumulative daily fluid balance prior to RRT	mls
Creatinine, urea, pH, bicarbonate, potassium on day of RRT initiation	result
Renal Recovery at ICU Discharge	y/n - IHD
Renal Recovery at Hospital Discharge	y/n – IHD/PD
Renal Recovery at 6 Months	y/n - IHD/PD
ICU Mortality	A/D
Hospital Mortality	A/D
6-month Mortality	A/D
ICU length of Stay	days
Hospital Length of Stay	days
Number of admissions to site	aggregate
Patient days	aggregate
Ventilator days	aggregate
RRT days	Days CRRT/IHD/SLED
CRRT and IRRT data - filter life, reasons for retiring filters, treatment time lost, prescription/dose, machine alarms, machine down times, type of coagulation, blood flow rates, filtration fraction, adverse events, solute clearance, ultrafiltration realized	aggregate
Economic data - cost of filters, fluids, anticoagulation medications, RRT catheters, patient life-years gained, quality of life adjusted years (QUALY), re-hospitalizations, recurrence/chronic RRT, health care provider related costs	aggregate
QOL and PROMs	aggregate

Data sources will include multiple Alberta Health Services administrative databases, the Nephrology Information System (NIS) and the Patient Based Renal Information System (PARIS) (Supplementary Table 1) [20, 21].

### Co-enrollment

Co-enrollment into other clinical research studies will be evaluated on a case-by-case basis.

### Statistical analyses

#### Health outcome measures

The patients’ characteristics and the target clinic outcomes in the baseline and intervention periods will be summarized. Mean (SD) and/or median (IQR) will be used for continuous variable; frequency will be used for categorical variables. To compare pre- and post-intervention difference, *p*-values will be provided by t-test (for normally distributed variables), non-parametric Wilcoxon tests (for non-normally distributed variables) or Chi-square test (for categorical variables). Interrupted time series (ITS) analyses will use autoregressive integrated moving average (ARIMA) models to determine changes in the KPI performance following the implementation of the acute KRT pathway.

#### KPI and interrupted time series analysis

Interrupted time series (ITS) analyses will be done using autoregressive integrated moving average (ARIMA) models to account for temporal trends and to determine whether there were changes in the process and clinical outcomes at the intervention period (compared with the baseline period) associated with implementation of the evidence-based acute KRT pathway. Each KPI will be assessed separately, as well as in aggregate with other KPIs. Autocorrelation, partial autocorrelation, and inverse autocorrelation functions will be assessed for model parameter appropriateness and seasonality. Stationarity will be assessed using the autocorrelation function and the augmented Dickey–Fuller test. The presence of ‘white noise’ was assessed by examining the autocorrelations at various lags, using the Ljung–Box χ2 statistic.

#### Healthcare system costs analysis

The primary health economic evaluation will be a within-study analysis of the cost-effectiveness of the Dialyzing Wisely program. Subject to available resources we will consider additional model-based analyses of Dialyzing Wisely over a longer time horizon.

The within-study analysis will be conducted on resource use and outcomes occurring during the study period. It will include total quarterly acute KRT-associated costs for each specific ICU following the implementation of KPI reporting. The cost analysis will include 1) utilization costs of CKRT filters, CKRT fluids, KRT anticoagulation (if any),and KRT catheters. Costs will be calculated in part using acute KRT process measures captured by our acute KRT KPIs.

We will also conduct an analysis of healthcare systems costs including those associated with total ICU and hospital stay and ongoing new ESKD costs (i.e., long-term, maintenance dialysis costs, total healthcare costs. Healthcare system costs will be reported as costs of acute KRT in ICU as a proportion of total ICU costs per quarter. Modeling analysis will capture costs to the health service, social care providers and patients so as to provide cost estimates from a societal perspective. We will determine models for averted and delayed acute KRT and averted new end-stage kidney disease requiring chronic dialysis based on observed changes in practice based on prescriber reports and adherence to best-evidence. Results will be reported as the incremental net benefit and incremental cost-effectiveness ratios. Uncertainty will be captured in the analyses through probabilistic sensitivity analysis and reported using cost- effectiveness acceptability curves, showing the likelihood the intervention will be cost-effective over a range of values of willingness-to-pay thresholds for specific outcomes.

#### Health-related quality of life and patient-reported outcome measures

Cost-effectiveness will be analyzed by estimating incremental cost and effectiveness based on, patient life-years gained and quality-adjusted life years [QALY]) gained. These will also be modeled based on adherence to best evidence-based practices and anticipated outcomes. QALYs will be calculated based on health-related quality of life as measured by the EQ-5D-5L and the PedsQL in children.

PROMs analysis will be conducted by quality-of-life assessment using the ESAS-r and/or the IPOS-renal scales [22]. This data will be collected at first chronic dialysis session. Quantitative analysis will be done to assess changes in scores using a linear mixed effects model with the baseline score as a fixed effect co-variate, and dialysis unit, with each cluster as a random intercept.

All statistical analyses will be done using SAS Enterprise Guide 7.1 (Cary, NC), TreeAge Pro (TreeAge Software Inc., Williamstown MA) and Excel (Microsoft, Redmond VA).

### Subgroup analysis

Pre-specified subgroup analysis will include ICU patients to 1) adult vs. children, 2) female vs. male, 3) academic vs. community/regional ICUs, 4) cardiovascular ICUs vs. Cardiac ICU vs. medical/surgical ICUs, 5) high KRT volume vs. low KRT volume centers (i.e., as per quartiles).

We will perform the above analyses for health economic evaluations, patient and process of case measures to include our pre-specified primary and secondary outcomes for each subgroup. Each analysis will be accompanied by a test for interaction between treatment and subgroup to ascertain whether effects differ significantly between subgroups.

### Ethics approval and consent to participate

This evaluation was reviewed by the University of Alberta Health Research Ethics Board (HREB) and a waiver of consent was granted based on the premise this project represents health services implementation and evaluation compatible with a quality assurance and improvement initiative.

### Knowledge translation and dissemination

Dialyzing Wisely is largely a program focused on knowledge translation and implementation of an acute KRT pathway based on evidence-based best practices.

Throughout the project period Clinical Practice Leads will review sub optimally performing KPIs across acute KRT programs and provide targeted education to individual ICU teams to best understand opportunities to drive improved performance. The Clinical Practice Leads will also facilitate the implementation of evidence-based best practices into clinical practice through individualized prescriber reports regarding acute KRT initiations and bedside provider education.

We will publish two peer-reviewed manuscripts for the Dialyzing Wisely program, one outlining the protocol, and the second disseminating study results. Additional manuscripts may be developed to present subgroup findings as well as patient-centered outcomes including PROMs. In addition, the results of the Dialyzing Wisely program will be presented at local, provincial, and national critical care and nephrology meetings.

## Discussion

In Alberta, we do not routinely measure when we initiate acute KRT, or how well we perform this therapy in ICU settings. We recognize there are KPIs that can be implemented that are not used routinely in clinical practice [9, 23, 24]. These are missed opportunities to improve the quality and safety of care, reduce unnecessary practice variation and address an important evidence-to-care gap on one of the key life-support technologies used in ICU [23–26].

Using the Quality Enhancement Research Initiative (QUERI) Roadmap for Implementation and Quality Improvement, the Dialyzing Wisely program will use a pragmatic strategy on how to adopt, adapt, implement, spread and sustain these evidence based clinical practices and innovations across an entire healthcare system [27]. This pathway will use the Learning Health System Knowledge to Action Framework with the principles of robust pre-implementation planning, followed by appropriate implementation strategies to ensure the sustainability of the program after the transition of ownership of the program to local stakeholders [16]. Having the implementation of the acute KRT pathway follow the roll-out of our CIS will ensure that the program will continuously seek to generate and learn from local data in order to improve systems and individual performance and enhance individual health and quality of care [28, 29]. To date, care gaps have been identified and a solution determined based on recently published evidence. Local stakeholders have been engaged and consulted in the development of the pathway. Evidence-based change techniques will be utilized to enact change across units, adapting to local policies and workflows to ensure the seamless integration of the acute KRT pathway into each ICU [30]. Our study team has previously embarked on a similar program of work, QUALITY CRRT, which aims to improve solely the performance of CKRT in our ICUs [31]. Dialyzing Wisely will expand on this work to not only evaluate KPI’s for CKRT, but for the provision of IKRT and prescriber practices as well in order to transform the delivery of acute dialysis throughout the critical care healthcare system.

The Dialyzing Wisely program will build on the infrastructure within our CIS to develop a easy and simple way for monitoring KPIs, to report progress to stakeholders and make data accessible to each ICU. Finally, to transition ownership to each ICU and associated stakeholders, we will work with our partner organizations to ensure the appropriate management support for each program to support continuous learning from knowledge gained throughout the Dialyzing Wisely initiative.

## Limitations

Dialyzing Wisely looks to improve the performance of acute KRT. However, this program does not address the care of patients with pre-existing chronic kidney disease (CKD) with dialysis initiated as part of a long term management plan. These patients are the majority of patients with new ESKD who enter long term chronic dialysis programs. We will share the experience gained in Dialyzing Wisely with other teams managing ESRD in order to facilitate and promote the monitoring of KPIs in this patient population.

Finally, the discontinuation or modality choice of acute dialysis is not part of the Dialyzing Wisely acute dialysis pathway. This is in large part due to the lack of evidence-based protocols for weaning and discontinuation from acute KRT, or transition from CKRT to IKRT. However, Dialyzing Wisely will build a framework that can be utilized for the implementation of new evidence across our ICUs and acute dialysis prescribers once it becomes available.

## Conclusion

Dialyzing Wisely will implement an acute dialysis care pathway across Alberta ICUs in order to improve the performance of acute dialysis, ensure prescribers follow best-evidence based practices, and decrease healthcare system costs while improve the quality of lives of people living in Alberta.

## Supplementary Information

**Additional file 1: Supplementary Table 1.** Outline of data sources

**Additional file 2.**

## Data Availability

Not applicable.
