# Supplementary material for: A study protocol for improving the delivery of acute kidney replacement therapy (KRT) to critically ill patients in Alberta – DIALYZING WISELY
Source: BMC Nephrol. 2022 Nov 16;23:369. doi: 10.1186/s12882-022-02990-6 (PMC9670635; doi:10.1186/s12882-022-02990-6)
Supplement: Supplementary file 1 — Additional file 1: Supplementary Table 1. Outline of data sources [file 12882_2022_2990_MOESM1_ESM.docx]

**Supplementary table 1: Alberta Health Services databases**

| **Data Repository** | **Description** |
| --- | --- |
| Connect Care/Enterprise, eCritical/TRACER | Province-wide ICU clinical data including demographic, diagnostics, laboratory results, device outputs and interdisciplinary clinical documentation |
| Data integration, management and reporting (DIMR) | Province-wide administrative database including: discharge abstract database, ambulatory care reporting system, physician billing, medical laboratory, pharmaceutical information network, vital statistics and provincial registry |
| Nephrology Information System (NIS)  Patient based renal information system (PARIS) | Provincial renal databases including all chronic dialysis starts and chronic dialysis related metrics |
